# Multifocal Metastatic Breast Carcinoma to the Thyroid Gland Histologically Mimicking C Cell Lesions

**DOI:** 10.1155/2019/9890716

**Published:** 2019-03-10

**Authors:** Adeeba F. Ghias, Gregory Epps, Elizabeth Cottrill, Stacey K. Mardekian

**Affiliations:** ^1^Sidney Kimmel Medical College at Thomas Jefferson University, Philadelphia, PA 19107, USA; ^2^Department of Otolaryngology-Head and Neck Surgery, Thomas Jefferson University, Philadelphia, PA 19107, USA; ^3^Department of Pathology, Anatomy and Cell Biology, Thomas Jefferson University, Philadelphia, PA 19107, USA

## Abstract

The thyroid gland is an uncommon site of metastatic disease. Renal cell carcinoma is the most common primary source, while metastasis from breast carcinoma is very rare. However, given that thyroid nodules are more common in women, and women with a history of breast cancer are at higher risk of developing thyroid cancer, the possibility of metastatic breast carcinoma must be considered when evaluating a thyroid nodule. We present the case of a 67-year-old woman who presented with dysphonia and dysphagia secondary to multinodular goiter and was found to have multifocal metastatic breast carcinoma in her surgical resection specimen. The histologic appearance focally mimicked C cell hyperplasia and medullary thyroid carcinoma, so immunohistochemistry was critical for establishing the diagnosis. Metastasis to the thyroid should always be included in the differential diagnosis for a thyroid nodule in a patient with a history of previous malignancy.

## 1. Introduction

Metastasis to the thyroid from an extrathyroidal malignancy is an uncommon phenomenon, estimated to occur only in 1-3% of thyroid glands surgically removed for suspected malignancy [1, 2]. Of this small number, the most frequently reported primary tumor is renal cell carcinoma, which accounts for nearly half of all cases, followed by colorectal, lung, and breast carcinoma, sarcoma, and melanoma [1, 3]. The time interval between initial diagnosis of the primary malignancy and discovery of the thyroid metastasis varies widely, and tends to be shortest in patients with lung cancer (mean 4.5 months) and longest in patients with sarcoma (mean 75 months). Most patients present with symptoms related to a new or enlarging thyroid nodule, while approximately 25% of lesions are discovered incidentally. Abnormal thyroid glands affected by primary thyroid neoplasia, hyperplasia or thyroiditis, are hypothesized to have higher susceptibility to metastatic disease [1].

Despite breast cancer being the most commonly diagnosed cancer in women, metastasis to the liver, bones, and lungs is much more common than to the thyroid gland [4, 5]. Metastatic breast cancer is estimated to represent the fourth most common origin for metastasis to the thyroid [1]. A recent literature review found 42 cases of metastatic breast cancer to the thyroid reported between 1962 and 2012; invasive ductal carcinoma is more commonly implicated than invasive lobular carcinoma [6]. In postmortem studies, breast cancer is one of the most common primary sources of metastasis to the thyroid, suggesting that these metastases are more often clinically occult [2, 7, 8]. Moreover, women with a history of breast cancer are at higher risk of developing primary thyroid cancer, so a significant percentage of women presenting with thyroid nodules will possess a breast cancer history [9]. On fine needle aspiration (FNA), metastatic ductal breast carcinoma can mimic a primary thyroid carcinoma including papillary, follicular and medullary types [10]. However, immunohistochemical stains such as GATA3, PAX8, and TTF-1 can aid in differentiating metastatic breast carcinoma from primary thyroid carcinoma [2].

We present a case of metastatic breast cancer to a multinodular thyroid gland, in which the metastatic deposits histologically mimicked C cell lesions thus necessitating immunohistochemical staining for confirmation of diagnosis.

## 2. Case Presentation

### 2.1. Clinical History

A 67-year-old female presented with three months of hoarseness and dysphagia following an upper respiratory infection. Her past medical history included invasive ductal carcinoma of the breast (ER positive, PR negative, and HER2 negative) treated by mastectomy two years prior to presentation. On clinical examination, she was found to have paralysis of the right vocal cord, and a follow-up CT scan of the neck revealed an enlarged thyroid gland with multiple bilateral thyroid nodules. FNA of a right-sided 3.5 cm thyroid nodule was diagnosed as atypia of undetermined significance (Bethesda category III). A repeat FNA three months later yielded a diagnosis of benign follicular nodule (Bethesda category II). Persistent hoarseness and compressive symptoms, combined with atypical findings on the initial FNA, necessitated right thyroid lobectomy and right vocal cord injection. Intraoperatively, the right thyroid lobe was noted to be moderately enlarged with multiple nodules but no evidence of extrathyroidal extension.

### 2.2. Pathology

The right lobectomy specimen measured 4.6 cm in greatest dimension and weighed 16 grams. The cut surfaces of the thyroid parenchyma showed multiple variably sized brown gelatinous nodules, with focal areas of hemorrhage and cystic change.

**Figure 1:**
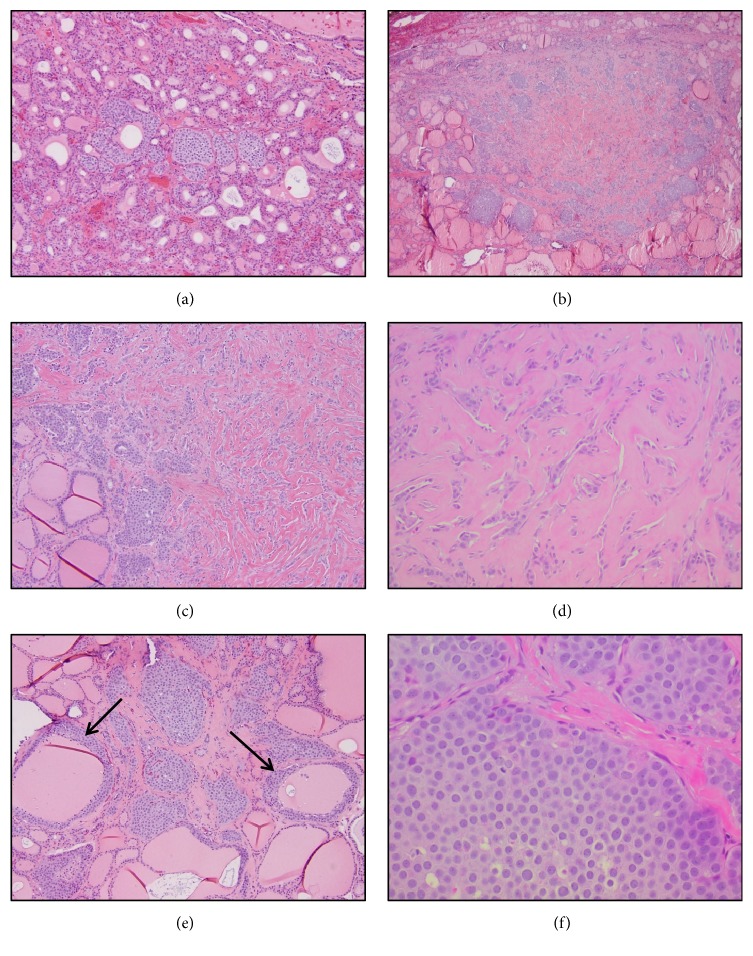
One of the smallest metastatic deposits consists of a few irregularly shaped nests of tumor cells spanning only one millimeter ((a), x100). One of the larger metastatic deposits consists of a fairly well-circumscribed proliferation of tumor cells, which are arranged peripherally in nests and centrally as cords within a sclerotic stroma ((b), x40). The densely sclerotic stroma in this focus of metastatic breast carcinoma resembles the amyloid-type stroma seen in medullary thyroid carcinoma ((c), x100), and the embedded cords and small nests of tumor cells display pronounced retraction artifact ((d), x200). Some metastatic deposits show prominent peripheral rimming of the thyroid follicles by tumor cells, in a pattern reminiscent of C cell hyperplasia ((e), x100). The tumor cells have round nuclei with fine chromatin and prominent nucleoli, ample eosinophilic cytoplasm, and distinct cell borders ((f), x400).

Histologic examination revealed evidence of nodular thyroid hyperplasia, along with multiple scattered subcentimeter foci of metastatic breast carcinoma, which were present in 7 out of the 16 submitted tissue sections. Most of the smaller metastatic foci, measuring around 1 millimeter each, consisted of a few irregularly shaped nests of epithelioid tumor cells interspersed between thyroid follicles (Figure 1(a)). The largest metastatic focus measured 0.6 cm and was comprised of tumor cell nests arranged along the periphery of a sclerotic stroma containing cords of tumor cells showing retraction artifact (Figures 1(b)–1(d)). These areas resembled the dense amyloid-type stroma often seen in medullary thyroid carcinoma (MTC). Additionally, there were numerous areas of rimming of the thyroid follicles by the tumor cells, which mimicked the appearance of C cell hyperplasia (CCH) (Figure 1(e)). Where arranged as nests, cell borders were distinct between the tumor cells, which contained centrally placed monomorphic round nuclei, finely granular chromatin, prominent nucleoli, and a moderate amount of eosinophilic cytoplasm (Figure 1(f)).

**Figure 2:**
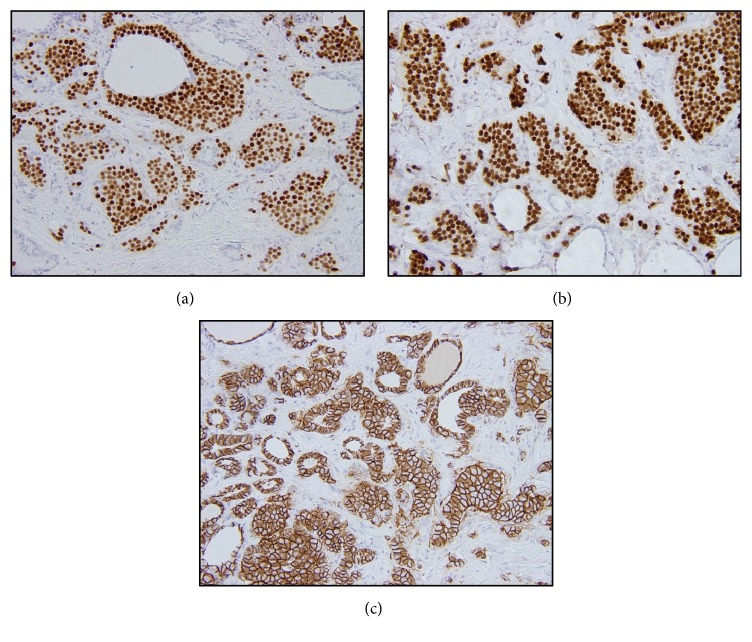
The tumor cells express nuclear positivity for ER ((a), x200) and GATA3 ((b), x200) immunohistochemistry, and diffuse membranous staining with e-cadherin ((c), x200).

Immunohistochemical stains showed that the tumor cells were positive for cytokeratin AE1/AE3, ER, GATA3, and e-cadherin (Figure 2), while they were negative for CK7, CK20, GCDFP/mammaglobin, TTF-1, thyroglobulin, calcitonin, and synaptophysin. Biomarker testing was performed and was scored by image analysis; the metastasis was ER positive (91.2%; moderate staining), PR positive (1.4%; weak staining), and HER2 negative (score 0 by IHC).

### 2.3. Clinical Follow-Up

At the one-week postoperative visit, the patient complained of right-sided hearing loss which had started the day of her surgery, as well as a two-day history of right-sided facial weakness. Audiologic testing showed a profound right-sided sensorineural hearing loss. Given these acute changes, she was directly admitted to the hospital for further workup where a brain MRI showed a right cerebellopontine angle mass concerning for further metastasis. A lumbar puncture was negative for malignancy; therefore, the patient underwent a retrosigmoid craniotomy for tissue diagnosis which confirmed metastatic breast cancer. Due to her extensive disease, no further surgical intervention was pursued.

## 3. Discussion

The thyroid gland has a rich arterial supply, yet it is a rare site of metastatic disease. This paradox is attributed to a combination of fast arterial flow hindering tumor cell adhesion, and high oxygen saturation and iodine content inhibiting tumor cell growth [1]. The clinical detection rate of metastatic lesions to the thyroid may be increasing with more accurate imaging and diagnostic techniques [6]. In a literature review of 374 metastatic lesions in the thyroid gland, Chung et al. [1] found that almost half of metastases to the thyroid gland occurred in diseased thyroids affected by primary thyroid neoplasia, goiter, or thyroiditis. These abnormal thyroid glands may be more susceptible to metastatic disease, perhaps due to aberrant blood supply resulting in reduced oxygen and iodine levels; however, metastatic disease appears to affect otherwise normal thyroid glands at an equal predilection [1].

Although breast cancer ranks fourth among clinically detected metastases to the thyroid, autopsy studies suggest it may actually be more common [1, 7, 8]. Detection of a metastatic lesion in the thyroid can occur as late as 12 years after treatment for the primary breast cancer, and is generally associated with poor prognosis [4, 7]. Most patients with clinically evident thyroid metastases have widespread metastatic disease, but occasionally the thyroid may appear to be the only metastatic site [6]. Surgical resection may not be indicated for intrathyroidal breast cancer metastases, so upfront detection is crucial if unnecessary surgery is to be avoided [4]. Unfortunately, sampling error may hinder the FNA diagnosis of metastatic lesions in the thyroid, especially in the setting of a large goitrous gland containing microscopic metastatic foci. Furthermore, metastatic lesions in the thyroid lack specific radiologic features that can reliably distinguish them from primary thyroid cancers, and their radiologic variability may reflect differences in the primary site of origin [11]. The ultrasonographic features of intrathyroidal breast cancer metastases may reportedly mimic primary thyroid malignancies such as papillary thyroid carcinoma (PTC), and some cases may not manifest as a discrete nodule but rather as diffuse calcifications within heterogeneous thyroid parenchyma [5, 7]. In our case, two episodes of FNA sampling targeted a 3.5 cm nodule which was a component of the patient's nodular thyroid hyperplasia. The subcentimeter metastatic foci were not sampled because whether or not they were radiologically apparent, thyroid lesions less than 1 cm are not routinely sampled by FNA.

If a metastatic lesion is successfully sampled via FNA, the most helpful clue to the diagnosis is the presence of a second distinct population of malignant cells that differ morphologically from follicular cells or the characteristic PTC nuclei [12]. However, metastatic tumor cells may cytologically mimic those of a primary thyroid neoplasm or even parathyroid tissue [10]. The distinction between MTC and a metastatic lesion in a thyroid FNA specimen can be especially challenging, as MTC cells can look epithelioid, plasmacytoid or spindled, and can be arranged in clusters or as many discohesive single cells [6, 12]. When analyzing a surgical specimen, tumor architecture is an additional feature that may be helpful in the differential diagnosis, but as demonstrated in our case, immunohistochemical stains are essential to arriving at a definitive diagnosis. In hereditary cases of MTC, patients often have more than one focus of primary tumor, and multifocal CCH is often present in the background thyroid parenchyma. In our case, there were multiple small metastatic deposits rimming the thyroid follicles and hence mimicking CCH, in addition to a few larger foci with dense stroma resembling the amyloid-type stroma seen in MTC. However, calcitonin and synaptophysin stains, which would be positive in the cells comprising MTC and CCH, were negative. A metastatic neuroendocrine carcinoma, from any site including the breast, to the thyroid would prove to be an especially challenging scenario, as it would not only morphologically mimic MTC but would also stain similarly with synaptophysin.

To exclude follicular-derived primary thyroid tumors, the stains thyroglobulin, TTF-1, and PAX8 are useful markers; however of these, thyroglobulin is the only specific marker of thyroid origin, as TTF-1 also stains lung cancer, and PAX8 stains renal and gynecologic tumors. Poorly differentiated metastatic tumors may pose a challenging differential diagnosis with anaplastic thyroid carcinoma, which by definition tends to lose expression of the traditional thyroid markers [5]. In our case, a positive pancytokeratin stain confirmed carcinoma, positive staining for the breast markers GATA3 and ER confirmed the site of origin, and membranous staining for e-cadherin supported a ductal phenotype. When considering breast cancer metastasis, GCDFP-15 and mammaglobin are other potential useful markers of breast origin, though they are less sensitive than GATA3 [10]. Furthermore, stains for ER, PR and HER2/neu are expected to stain similarly to the primary breast cancer if is known that the primary expressed any of these prognostic markers [13]. Interestingly, a study of 25 patients with metastatic breast cancer reported that ER positive tumors metastasized more frequently to thyroid and parathyroid glands than did ER-negative tumors, perhaps reflecting tissue differences in hormone-binding receptors [14].

Metastasis to the thyroid should be identified as early as possible, as there are differences in treatment protocols and prognosis when compared to primary thyroid cancers [12]. In one series, patients with a single metastatic lesion in the thyroid had significantly better survival than patients with multifocal metastatic disease [15]. Prognosis may also vary according to the primary site of origin and the histologic grade of the metastasis [3]. The role of surgery for metastatic lesions in the thyroid is unclear. Some studies suggest that patients managed surgically experience better outcomes than those managed expectantly [16]. In a study of metastatic renal cell carcinoma to the thyroid, median survival for those managed expectantly was 6 months versus 27 months for those who underwent surgery [17]. However, in the limited evidence pertaining to the management of breast cancer metastases of the thyroid, thyroidectomy does not appear to affect overall survival, which remains poor [2, 8, 18]. Nevertheless, large metastatic lesions may cause significant morbidity including dysphagia, dysphonia and airway compromise, so surgery may be warranted to alleviate these problems.

## 4. Conclusion

Although metastases of nonthyroid malignancies to the thyroid gland are rare, a high index of suspicion is important when managing a new thyroid nodule in a patient with a history of prior malignancy. The identification of metastatic breast carcinoma in a thyroid FNA or surgical specimen, aided by immunohistochemistry, may have important therapeutic and prognostic implications. We report the peculiar ability of metastatic breast carcinoma to mimic C cell lesions including CCH and MTC, which to our knowledge has not been previously described in the literature.
